# Efficacy and Tolerability of Intravenous Levetiracetam in Children

**DOI:** 10.3389/fneur.2013.00120

**Published:** 2013-08-14

**Authors:** Jose Aceves, Owais Khan, Diana Mungall, Ekokobe Fonkem, Chanin Wright, Andrea Wenner, Batool Kirmani

**Affiliations:** ^1^Department of Pediatrics, Division of Pediatric Neurology, Texas A&M Health Science Center College of Medicine, Scott & White Hospital, Temple, TX, USA; ^2^Department of Pediatrics, Division of Neonatology, University of Chicago Medical Center, Chicago, IL, USA; ^3^Texas A&M Health Science Center College of Medicine, Temple, TX, USA; ^4^Department of Neurology, Texas A&M Health Science Center College of Medicine, Scott & White Neuroscience Institute, Temple, TX, USA; ^5^Department of Pediatrics, Division of Pharmacy, Texas A&M Health Science Center College of Medicine, Scott & White Hospital, Temple, TX, USA; ^6^Department of Pediatrics, Texas A&M Health Science Center College of Medicine, Scott & White Hospital, Temple, TX, USA; ^7^Department of Neurology, Epilepsy Center, Texas A&M Health Science Center College of Medicine, Scott & White Neuroscience Institute, Temple, TX, USA

**Keywords:** epilepsy, intractable epilepsy, seizures, status epilepticus, intravenous levetiracetam, children

## Abstract

Intractable epilepsy in children poses a serious medical challenge. Acute repetitive seizures and status epilepticus leads to frequent emergency room visits and hospital admissions. Delay of treatment may lead to resistance to the first-line anticonvulsant therapies. It has been shown that these children continue to remain intractable even after acute seizure management with approved Food and Drug Administration (FDA) agents. Intravenous levetiracetam, a second-generation anticonvulsant was approved by the FDA in 2006 in patients 16 years and older as an alternative when oral treatment is not an option. Data have been published showing that intravenous levetiracetam is safe and efficacious, and can be used in an acute inpatient setting. This current review will discuss the recent data about the safety and tolerability of intravenous levetiracetam in children and neonates, and emphasize the need for a larger prospective multicenter trial to prove the efficacy of this agent in acute seizure management.

## Introduction

The pediatric epilepsy population is frequently admitted to the hospital because of status epilepticus or acute repetitive seizures. Status epilepticus is defined as continuous seizure activity lasting for 30 min or longer or intermittent seizures lasting for more than 30 min from which the patient does not regain consciousness (1). The other definition is a continuous seizure lasting at least 5 min, or two or more seizures without full recovery of consciousness between seizures lasting at least 5 min or more (2). Acute repetitive seizures are described as seizures that recur over a set period. These typically last for hours in children and up to 1 or 2 days in adults (3).

Acute management of seizures is crucial in the prevention of permanent neurological sequelae. Despite the use of benzodiazepines and first generation anticonvulsants, patients still remain refractory to treatment, emphasizing the need of newer anticonvulsants such as levetiracetam.

Levetiracetam is a pyrrolidine-derivative antiepileptic drug, which is chemically different from all other anticonvulsant agents. It has a novel mechanism of action, which does not involve inhibitory and excitatory neurotransmission. Levetiracetam works by binding SV2A, an integral membrane protein present on synaptic vesicles, preventing synaptic vesicle release (4). Thereby, impeding conduction across the synapse. The United States Food and Drug Administration (FDA) approved intravenous levetiracetam in August 2006 for patients above 16 years of age when oral treatment is not feasible.

Studies have demonstrated that intravenous levetiracetam has a favorable safety and pharmacokinetic profile as seen with oral levetiracetam in adult subjects (5, 6). Weinstock et al. conducted an Open-Label, Single-Arm, Multicenter, Safety, Tolerability, and Pharmacokinetic Study of Intravenous Levetiracetam in Children with Epilepsy. The age ranged from 1 month to 16 years of age. Fifty-two subjects were enrolled. No significant adverse events were reported and it was well tolerated in this population (7).

Levetiracetam has been found to be effective in certain experimental models of status epilepticus (8, 9). It also has been demonstrated that intravenous levetiracetam can be used as an alternative to oral dosing in patients, and data have been published about efficacy and safety in children of different age groups with epilepsy (10). In this review, we will discuss the literature that has displayed efficacy of intravenous levetiracetam in children and neonates.

### Efficacy in neonates

Seizures affect approximately 1–5 out of 1000 newborns and 11 out of 1000 preterm neonates (11–13). The most common causes of neonatal seizures include hypoxic-ischemic encephalopathy, intracranial hemorrhages, infections of the central nervous system, cerebral infarctions, and metabolic disturbances (11, 12). Protection and prevention of significant adverse effects upon the developing brain is critical in neonatal period. Phenobarbital, which acts via GABAergic mechanisms, remains as the most frequently used antiepileptic drug for the treatment of neonatal seizures. Current research suggests that the GABA receptor may be prone to deficient inhibition in the neonate (11). A muted or net excitatory affect from the binding of GABA in the neonatal brain may be due to an increased concentration of chloride in the immature brain’s neurons (11). Additionally, evidence exists that phenobarbital causes neuronal apoptosis in animal models, and long term adverse neurodevelopmental effects related to phenobarbital have been demonstrated (14). Several other antiepileptic drugs are being researched and prescribed in children and neonates (14, 15). Levetiracetam is increasingly being used as an antiepileptic drug in the neonatal period, and is recognized as an antiepileptic drug with neuroprotective properties (14, 16). Koppelstäetter et al. reported on the use of levetiracetam in term and preterm neonates with rarely observed adverse effects in their analysis of surveys from neonatologists and pediatric neurologists (17). A study by Kilicdag et al. demonstrated a significant decrease in the number of apoptotic neuronal cells in a levetiracetam treated group of rat pups who underwent a hypoxic-ischemic brain injury (16).

In the only randomized control trial of antiepileptic drugs in neonates, Painter et al. demonstrated efficacy of seizure cessation in less than 50% of patients treated with phenobarbital and phenytoin. Phenobarbital and phenytoin were used to treat a variety of neonatal seizure etiologies in these studies, with the majority of patients having underlying hypoxic-ischemic encephalopathy (18). Retrospective studies have demonstrated that intravenous levetiracetam may be efficacious in the management of acute seizures in neonates when other medications have failed (19, 20).

Abend et al. conducted a retrospective cohort study of 23 neonates in the Newborn Infant Intensive Care Unit with electro-clinical or electrographic-only confirmed seizures who received LEV. There were 12 female and 11 males with a mean gestational age of 38.7 ± 1.7 weeks. Patients were identified using the electronic pharmacy database over a 1-year period. IV LEV bolus doses of 10–20 mg/kg were given to neonates. Next, LEV was administered twice per day. LEV effectiveness was defined as a greater than 50% reduction in electrographic seizure within 24 h of the start of treatment. Neonatal seizure etiologies included eight hypoxic-ischemic encephalopathy, four presumed genetic/metabolic disorders, three brain malformations, three central nervous system infections, two strokes, two cryptogenic seizures, and one tumor. Seizure types included focal or multifocal clonic or tonic (18/23), subtle seizures (5/23), and desaturation/apnea (2/23). LEV was started at a mean age of 41 weeks. The mean initial dose was 16 ± 6 mg/kg and the mean maximum dose was 45 ± 19 mg/kg/day. There were no reported or detected respiratory or cardiovascular adverse effects. Greater than 50% seizure reduction within 24 h was considered to be effective and LEV was effective in 8 of 23 (35%). Seven of 23 patients had a complete seizure resolution (19).

Khan et al. performed a retrospective chart review of electronic medical records for neonates treated with IV LEV between January 2007 and December 2009. The researchers identified 22 neonates (0–28 days of age) born at term (≥37 weeks) with neonatal seizures who received IV LEV. Loading doses were from 10 to 50 mg/kg (20 patients received a loading dose of 50 mg/kg). Seizure etiologies included hypoxic-ischemic encephalopathy (12/22, 55%), hemorrhage (2/22, 9%), viral meningoencephalitis (2/22, 9%), and one patient each with benign neonatal seizures, brain malformation, cryptogenic partial seizures, glucose transporter protein type 1 deficiency, infarction, and trauma (1/22, 5%). Nineteen of 22 patients (86%) demonstrated immediate seizure cessation within 1 h. After loading dose complete seizure cessation was achieved in 7 of 22 patients (32%), by 24 h in 14 of 22 (64%), by 48 h by 19 of 22 (86%), and by 72 h in all 22 (100%). The authors concluded that IV LEV can be used in neonates as monotherapy and adjunct therapy in acute seizure management (20).

Levetiracetam continues to be used in a variety of clinical situations and seizure etiologies in neonates. Shoemaker and Rotenberg reported on the successful use of levetiracetam in neonates with varying seizure etiologies (21). Hmaimess et al. showed the efficacy of levetiracetam in a neonate with intractable malignant migrating partial seizures (22). Ledet et al. also showed efficacy as a prophylactic antiseizure medication in a neonate with acute lymphoblastic leukemia (23).

Furwentsches et al. examined the use of LEV as monotherapy in the treatment of neonatal seizures in a prospective pilot feasibility study. The study sample size included six consecutive mature or premature newborns presenting with neonatal seizures. Exclusion criteria included seizures due to electrolytes or hypoglycemia, seizures who did respond to pyridoxine, and patients previously treated with other AEDs. LEV was administered orally over 3 days with each dose increasing 10 mg/kg/day up to 50 mg/(kg/day). The study endpoint was either if the patient needed additional AEDs after day 3 or 3 months of LEV treatment. Seizure semiology major symptoms include tonic and apnea (3/6 patients, 50%), oral automatisms, staring and apnea (2/6 patients, 33%), and clonic and apnea (1/6, 17%). The researchers did not observe any severe adverse effects, although one infant had mild sedation. Within 6 days, all six patients treated with oral LEV became seizure free. Three months later, after ongoing LEV monotherapy, five patients remained seizure free. The remaining patient did not respond to therapy and developed pharmacoresistant epilepsy (24).

IV LEV was used as a first-line treatment in Ramantani et al.’s prospective feasibility study. From 2006 to 2008, the study consisted of 38 consecutively admitted newborns with EEG-confirmed seizures and excluded seizures due to electrolyte deficiencies and pyridoxine dependency. Nineteen of 38 newborns were extremely premature at gestational age<28 weeks. Seizure semiology included subtle, focal clonic, multifocal clonic, focal tonic, generalized tonic, and myoclonic. Patients<28 weeks gestational age most commonly had subtle seizure semiology (*N* = 12, 63%), patients 28–36 weeks most commonly had multifocal clonic seizures (*N* = 4, 67%), and patients ≥37 weeks most commonly had focal clonic seizures (*N* = 5, 38%). Intravenous doses of LEV were started at 10 mg/kg and over the course of 3 days were increased to 30 mg/kg, and at the end of the week were further titrated to 45–60 mg/kg. When the infant’s condition allowed, IV LEV was switched to oral administration. Up to two IV doses of phenobarbital (20 mg/kg) during LEV titration were tolerated for acute intervention. Acute interventions of one dose of phenobarbital was needed in 19 patients and three patients required two doses. At the conclusion of the first week, 30 infants treated with LEV were seizures free, while at the end of 4 weeks 27 infants remained seizure free. Seven infants received LEV for a duration up to 3 months, but in 19 infants LEV was discontinued after 2–4 weeks. There was no report of severe adverse effects. Ramantani et al. concluded that the results indicated the safety and efficacy of LEV treatment in neonatal seizures (25).

Pharmacokinetic studies have established a benign safety profile for levetiracetam. Merhar et al. used initial loading doses of 15–40 mg/kg, and demonstrated that levetiracetam was well tolerated in 18 neonates with seizures. The only adverse event present was somnolence. The study also found linear kinetics, minimal protein binding, and no hepatic metabolism with levetiracetam use in neonates (26). At this time, there are no clear dosing recommendations available in the literature where doses range from 10 to 70 mg/kg (15, 19, 20, 26–29).

### Efficacy in children

There have been multiple case reports, case series, and retrospective studies reported in children which showed the efficacy and tolerability of intravenous levetiracetam in acute seizure management both as adjunctive and monotherapy.

Haberlandt et al. showed the use of levetiracetam in the treatment of two children with myoclonic status epilepticus (30). Alehan et al. contributed a case report to the literature supporting the use of levetiracetam in children with non-convulsive status epilepticus (31). Weber et al. showed efficacy of levetiracetam in a child with Angelman syndrome presenting in non-convulsive status epilepticus (32). Cilio et al. showed the termination of refractory status epilepticus in two patients with migrating partial seizures in infancy with intravenous levetiracetam (27).

Michaelides et al. performed a retrospective analysis of a pediatric population under the age of 14 on the use of IV formulation of LEV for in its first 9 months of availability in their institution. The researchers reviewed the paper and electronic medical records of 15 children ≤14 years of age (3 months–14 years) who received IV LEV in the first 9 months that it was available at the institution. Seizure etiologies included six patients had complex partial seizures, three had generalized tonic-clonic, two no seizure type only for prophylaxis, one myoclonic, one tonic, and one mixed. Over the 9 months there were 118 infusions performed in 15 patients. No adverse reactions were observed. Nine minor adverse reactions were observed during the rest of the hospitalization that were potentially related to the IV formulation: three of these were decreases in WBC count, and six were behavioral adverse effects. In LEV naïve patients (*N* = 7) the median starting dose was 8.8 mg/kg (range: 5.0–50), and all patients had a median maintenance dose of 30.4 mg/kg/day (5.0–92). There were six patients in a subgroup less than 4 years old (avg. = 1 year 4 months), who received the majority of the LEV infusions in the entire study population (82/118 total infusions). Of the 15 patients, five had very frequent seizures (two with status epilepticus) and three of these patients had a seizure frequency reduction of>50%. One patient had complete seizure resolution. Michaelides et al. concluded that IV LEV was very well tolerated in their pediatric population (28).

Goraya et al. retrospectively identified 10 patients (aged 3 weeks–19 years) through their hospital pharmacy records all patients who received intravenous levetiracetam. Forty percent of patients had received IV levetiracetam for acute repetitive seizures/status epilepticus, 30% presented on levetiracetam, 10% received IV LEV for seizure prophylaxis for a brain biopsy, 10% received it for severe thrombocytopenia, and 10% for an acute symptomatic seizure. The dosages of IV LEV used varied according to the indication for usage: replacement of oral LEV used a mg for mg substitution, for status epileptics 20–40 mg/kg/dose was used every 8 h for infants or 12 h for older children, and for maintenance treatment after biopsy a dose of 10–20 mg/kg every 12 h was used and was given as an infusion. Seventy-five percent of the patients who received IV LEV for control of status epilepticus (three of four) became seizure free and 25% (one of four) had a>50% reduction in seizure frequency. This neonatal patient became seizure free on IV LEV after an area of cortical dysplastic tissue that had been found was removed. None of the 10 patients had any adverse events noted during IV LEV usage and there was no discontinuation of IV LEV due to side effects (29).

Kirmani et al. retrospectively reviewed 32 pediatric patients from 2 months to 18 years of age. The sample size included 53.1% males and 46.8% females. Data were acquired from electronic medical records for patients admitted in the hospital with status epilepticus or acute exacerbation of seizure patients who received intravenous levetiracetam. The loading dose used was 25–50 mg/kg. Data analysis showed a favorable response to intravenous levetiracetam for all patients and seizures were aborted both clinically and electrographically. In 18 patients, intravenous levetiracetam was infused after fosphenytoin and lorazepam. No serious side effects were reported in all subjects. Fifteen patients were discharged on levetiracetam monotherapy and nine on adjunctive therapy. The study concluded that intravenous levetiracetam was found to be efficacious both in status epilepticus and acute exacerbation of seizures (33).

Abend et al. described a cohort of critically ill children who received intravenous levetiracetam for status epilepticus or acute repetitive seizures. All the subjects responded to intravenous levetiracetam resulting in either termination, temporary cessation, or reduction in ongoing seizure activity (34). Khurana et al. conducted a retrospective analysis at a single institution over a period of 3 years. Their group identified 81 patients, in which 18 of them received levetiracetam as monotherapy. Fourteen patients had partial epilepsy and four had generalized epilepsy. The dose range was 14–60 mg/kg, and duration of therapy ranged from 2 to 24 months. The study concluded that levetiracetam was found to be efficacious as monotherapy in the management of pediatric epilepsy (35).

Gallentine et al. conducted a retrospective analysis over a 7-year period. Eleven children had received levetiracetam for refractory status epilepticus with the age ranging from 2 days to 9 years. The patients were treated with two to seven anticonvulsants prior to infusion of levetiracetam. Levetiracetam was found to be efficacious in 45% of cases, resulting in resolution of refractory status epilepticus. The median latency to resolution of status epilepticus following initiation of levetiracetam was 1.5 days. The responding subjects received a median dose 40 mg/kg/day. No significant adverse effects were reported. The authors concluded that levetiracetam was safe to use as adjuvant therapy in children with refractory status epilepticus (36).

Pharmacokinetic studies have established a benign safety profile for levetiracetam. Li et al. demonstrated that levetiracetam is a safe and effective treatment for infants and children in an observational, prospective study Li et al. prospectively analyzed 120 patients (39.3% female, 61.7% male) with epilepsy receiving mono or combination therapy with levetiracetam that were 14-year-old and younger. Therapy was started with a levetiracetam dose of 10 mg/kg/day and if seizures were poorly controlled titrated up with 10 mg/kg increments to a maximum daily dose of 60 mg/kg/day. They documented seizure type, seizure frequency, levetiracetam dose, and side effects. Li et al. found that the 83.0% of the study population had a seizure reduction of at least 50%, and 54.8% of patients were seizure free. There was a side effect incidence rate of 47.5%, and included somnolence, dysphoria, nervousness, dystrophy, somnipathy, astitia, and debilitation. There was a 3.3% patient withdrawal rate most commonly due to poor effect or intolerance of side effects. The researchers concluded that levetiracetam was safe and effective for epileptic children with multiple types of epileptic seizures (37).

## Conclusion

Linear kinetics, minimal protein binding, and no hepatic metabolism with levetiracetam use in children and neonates, and favorable response in status epilepticus and repetitive seizures makes levetiracetam a suitable choice in acute seizure management in both children and neonates. However, larger prospective, randomized, blind comparison trials between phenobarbital, levetiracetam, and phenytoin would provide more information regarding the use of this newer agent in acute management of seizures in the pediatric population.

## Conflict of Interest Statement

The authors declare that the research was conducted in the absence of any commercial or financial relationships that could be construed as a potential conflict of interest.

## References

[1] Treatment of convulsive status epilepticus. Recommendations of the Epilepsy Foundation of America’s Working Group on Status Epilepticus JAMA (1993) 270:854–9 10.1001/jama.270.7.854 8340986

[2] Lowenstein DH Bleck T Macdonald RL It’s time to revise the definition of status epilepticus. Epilepsia (1999) 40:120–2 10.1111/j.1528-1157.1999.tb02000.x 9924914

[3] Mitchell WG Status epilepticus and acute repetitive seizures in children, adolescents, and young adults: etiology, outcome and treatment. Epilepsia (1996) 37(Suppl l):S74–80 10.1111/j.1528-1157.1996.tb06025.x 8647055

[4] Lynch B Lambeng N Nocka K Kensel-Hammes P Baijalieh SM Matagne A The synaptic vesicle protein SV2A is the binding site for the antiepileptic drug levetiracetam. Proc Natl Acad Sci U S A (2004) 101:9861–6 10.1073/pnas.0308208101 15210974 PMC470764

[5] Ramael S Daoust A Otoul C Toublanc N Troenaru M Lu ZS Levetiracetam intravenous infusion: a randomized, placebo-controlled safety and pharmacokinetic study. Epilepsia (2006) 47:1128–35 10.1111/j.1528-1167.2006.00586.x 16886975

[6] Ramael S De Smedt F Toublanc N Otoul C Boulanger P Riethuisen JM Single-dose bioavailability of levetiracetam intravenous infusion relative to oral tablets and multiple-dose pharmacokinetics and tolerability of levetiracetam intravenous infusion compared with placebo in healthy subjects. Clin Ther (2006) 28:734–44 10.1016/j.clinthera.2006.05.004 16861095

[7] Weinstock A Ruiz M Gerard D Toublanc N Stockis A Farooq O Prospective open-label, single-arm, multicenter, safety, tolerability, and pharmacokinetic studies of Intravenous levetiracetam in children with epilepsy. J Child Neurol (2013). [Epub ahead of print]. 10.1177/0883073813480241 23533164

[8] Klitgaard H Matagne A Gobert J Wülfert E Evidence for a unique profile of levetiracetam in rodent models of seizures and epilepsy. Eur J Pharmacol (1998) 353:191–206 10.1016/S0014-2999(98)00410-5 9726649

[9] Mazarati AM Baldwin R Klitgaard H Matagne A Wasterlain CG Anticonvulsant effects of levetiracetam and levetiracetam-diazepam combinations in experimental status epilepticus. Epilepsy Res (2004) 58:167–74 10.1016/j.eplepsyres.2004.02.002 15120747

[10] Baulac M Brodie MJ Elger CE Krakow K Stockis A Mewisch P Levetiracetam intravenous infusion as an alternative to oral dosing in patients with partial-onset seizures. Epilepsia (2007) 48:589–92 10.1111/j.1528-1167.2006.00959.x 17326794

[11] Olson DM Neonatal seizures. Neoreviews (2012) 13:e213–23 10.1542/neo.13-4-e213

[12] Thibeault-Eybalin MP Lortie A Carmant L Neonatal seizures: do they damage the brain? Pediatr Neurol (2009) 40:175–80 10.1016/j.pediatrneurol.2008.10.026 19218030

[13] Ronen GM Penney S Andrews W The epidemiology of clinical neonatal seizures in Newfoundland: a population-based study. J Pediatr (1999) 134:71–5 10.1016/S0022-3476(99)70374-4 9880452

[14] Cilio MR Ferriero DM Synergistic neuroprotective therapies in hypothermia. Semin Fetal Neonatal Med (2010) 15:293–8 10.1016/j.siny.2010.02.002 20207600 PMC2892736

[15] Silverstein FS Ferriero DM Off-label use of antiepileptic drugs for the treatment of neonatal seizures. Pediatr Neurol (2008) 39:77–9 10.1016/j.pediatrneurol.2008.04.008 18639748

[16] Kilicdag H Daglioglu K Erdogan S Guzel A Sencar L Polat S The effect of levetiracetam on neuronal apoptosis in neonatal rat model of hypoxic ischemic brain injury. Early Hum Dev (2013) 89:355–60 10.1016/j.earlhumdev.2012.12.002 23266150

[17] Koppelstäetter A Buhrer C Kaindl AM Treating neonates with levetiracetam: a survey among German University Hospitals. Klin Padiatr (2011) 223:450–2 10.1055/s-0031-1287822 22105561

[18] Painter MJ Scher MS Stein AD Armatti S Wang Z Gardiner JC Phenobarbital compared with phenytoin for the treatment of neonatal seizures. N Engl J Med (1999) 341:485–9 10.1056/NEJM199908123410704 10441604

[19] Abend NS Gutierrez-Colina AM Monk HM Dlugos DJ Clancy RR Levetiracetam for the treatment of neonatal seizures. J Child Neurol (2011) 26:465–70 10.1177/0883073810384263 21233461 PMC3082578

[20] Khan O Chang E Cipriani C Wright C Crisp E Kirmani B Use of intravenous levetiracetam for management of acute seizures in neonates. Pediatr Neurol (2011) 44:265–9 10.1016/j.pediatrneurol.2010.11.005 21397167

[21] Shoemaker MT Rotenberg JS Levetiracetam for the treatment of neonatal seizures. J Child Neurol (2007) 22:95–8 10.1177/0883073807299973 17608315

[22] Hmaimess G Kadhim H Nassogne MC Bonnier C van Rijckevorsel K Levetiracetam in a neonate with malignant migrating partial seizures. Pediatr Neurol (2006) 34:55–9 10.1016/j.pediatrneurol.2005.06.011 16376281

[23] Ledet DS Wheless JW Rubnitz JE Brannon Morris E Levetiracetam as mono-therapy for seizures in a neonate with acute lymphoblastic leukemia. Eur J Paediatr Neurol (2010) 14:78–9 10.1016/j.ejpn.2008.12.007 19186085 PMC3902105

[24] Furwentsches A Bussman C Ramantani G Ebinger F Philippi H Poschl J Levetiracetam in the treatment of neonatal seizures: a pilot study. Seizure (2010) 19:185–9 10.1016/j.seizure.2010.01.003 20133173

[25] Ramantani G Ikonomidou C Walter B Rating D Dinger J Levetiracetam: safety and efficacy in neonatal seizures. Eur J Paediatr Neurol (2011) 15:1–7 10.1016/j.ejpn.2010.10.003 21094062

[26] Merhar SL Schibler KR Sherwin CM Meinzen-Derr J Shi J Balmakund T Pharmacokinetics of levetiracetam in neonates with seizures. J Pediatr (2011) 159:152–4 10.1016/j.jpeds.2011.03.057 21592494 PMC3789844

[27] Cilio MR Bianchi R Balestri M Onofri A Giovannini S Di Capua M Intravenous levetiracetam terminates refractory status epilepticus in two patients with migrating partial seizures in infancy. Epilepsy Res (2009) 86:66–71 10.1016/j.eplepsyres.2009.05.004 19520548

[28] Michaelides C Thibert RL Shapiro MJ Kinirons P John T Manchharama D Tolerability and dosing experience of intravenous levetiracetam in children and infants. Epilepsy Res (2008) 81:143–7 10.1016/j.eplepsyres.2008.05.004 18571898

[29] Goraya JS Khurana DS Valencia I Melvin JJ Cruz M Legido A Intravenous levetiracetam in children with epilepsy. Pediatr Neurol (2008) 38:177–80 10.1016/j.pediatrneurol.2007.11.003 18279751

[30] Haberlandt E Sigl SB Scholl-Buergi S Karall D Rauchenzauner M Rostasy K Levetiracetam in the treatment of two children with myoclonic status epilepticus. Eur J Paediatr Neurol (2009) 13:546–9 10.1016/j.ejpn.2008.09.006 19010072

[31] Alehan F Ozcay F Haberal M The use of levetiracetam in a child with nonconvulsive status epilepticus. J Child Neurol (2008) 23:331–3 10.1177/0883073807309237 18192652

[32] Weber P Levetiracetam in nonconvulsive status epilepticus in a child with Angelman syndrome. J Child Neurol (2010) 25:393–6 10.1177/0883073809338626 19605773

[33] Kirmani BF Crisp ED Kayani S Rajab H Role of intravenous levetiracetam in acute seizure management of children. Pediatr Neurol (2009) 41:37–9 10.1016/j.pediatrneurol.2009.02.016 19520272

[34] Abend NS Monk HM Licht DJ Dlugos DJ Intravenous levetiracetam in critically ill children with status epilepticus or acute repetitive seizures. Pediatr Crit Care Med (2009) 10:505–10 10.1097/PCC.0b013e3181a0e1cf 19325512 PMC2946960

[35] Khurana DS Kothare SV Valencia I Melvin JJ Legido A Levetiracetam monotherapy in children with epilepsy. Pediatr Neurol (2007) 36:227–30 10.1016/j.pediatrneurol.2006.09.018 17437904

[36] Gallentine WB Hunnicutt AS Husain AM Levetiracetam in children with refractory status epilepticus. Epilepsy Behav (2009) 14:215–8 10.1016/j.yebeh.2008.09.028 18926926

[37] Li J Xiao N Chen S Efficacy and tolerability of levetiracetam in children with epilepsy. Brain Dev (2011) 33:145–51 10.1016/j.braindev.2010.03.002 20359839

